# Establishment of reference range of CD4 T-lymphocyte in healthy Nepalese adults

**DOI:** 10.1186/s13104-020-05156-5

**Published:** 2020-07-02

**Authors:** Shravan Kumar Mishra, Lilee Shrestha, Roshan Pandit, Sundar Khadka, Bimal Shrestha, Subhash Dhital, Saroj Sharma, Mukunda Sharma, Raj Kumar Mahato, Geeta Shakya, Krishna Das Manandhar

**Affiliations:** 1 National Public Health Laboratory, Department of Health Services, Ministry of Health and Population, Kathmandu, Nepal; 2 Central Department of Biotechnology, Institute of Science and Technology, Tribhuvan University, Kathmandu, Nepal; 3 Kanti Children Hospital, Maharajgunj, Kathmandu, Nepal

**Keywords:** Reference range, CD4, CD3, T-lymphocyte, Nepal

## Abstract

**Objective:**

CD4 T lymphocytes are the most widely used cellular markers to assess the course of HIV infection, clinical staging and, monitoring the effect of antiretroviral therapy. The regional reference range for Eastern, Central and Western development region of Nepal had already been established whereas the same was still lacking in Mid-western and Far-western development region. The objective of this study was to establish reference range of CD4 T lymphocyte in the remaining two development regions and finally the national reference range using data from previous study.

**Results:**

The average values (mean ± SD) of CD4 and CD3 T cell in present study was (819 ± 294) cells/μl and (1546 ± 532) cells/μl, respectively. The absolute CD4 T cell (914 ± 303) and CD3 T cell (1671 ± 560) count in female were significantly higher than those from male, CD4 (757 ± 270) and CD3 (1465 ± 499) (p value-0.000). National reference value of CD4 was determined to be (798 ± 335) cells/μl for healthy Nepalese adults.

## Introduction

CD4 subset is the T lymphocyte sub-population that is commonly used in clinical arena for decision making of various conditions [1]. It is most widely used blood parameters to monitor human immunodeficiency virus (HIV) patients for disease progression, clinical staging, epidemiological studies, and prophylaxis of some opportunistic infection [2]. CD4 subset tends to decreases with disease progression, leading to relative increase of CD8 T lymphocyte and subsequent reversal of CD4 to CD8 T lymphocyte ratio [3]. The course of disease is better understood by comparing CD4 count with the available local reference range.

HIV RNA viral load testing is considered gold standard to monitor antiretroviral therapy (ART) response. Despite its high sensitivity, viral load testing has a number of problems in use and, is not feasible in all the settings due to high cost and technical demands [4]. In such scenario, CD4 T cell count, and clinical monitoring is recommended to monitor the efficacy of antiretroviral therapy, especially in resource-poor settings [5]. In addition, CD4 count may also be used in decision making for initiation of ART in HIV patients [6]. National HIV testing and treatment guideline in Nepal suggests CD4 count as one of the baseline test to monitor antiretroviral therapy that has to be checked every 6 months. The further steps on prophylaxis could be taken based on CD4 level along with some other baseline test such as Hemoglobin estimation, Urea and Alanine Aminotransferase [6]. These all show that CD4 count is necessary to guide antiretroviral therapy and, to decrease the chance for morbidity, mortality and vertical transmission of the disease [7].

CD4 count testing service was available at eighteen sites across the country till 2014, which reached to thirty by 2017 [8]. HIV RNA viral load testing was provided only from three centers: National Public Health Laboratory in Kathmandu, Bir Hospital in Kathmandu and Seti Zonal Hospital in Kailali to supplement the ART management program till 2017 [9]. As Nepal is a developing country with weak economic status, it is difficult to establish viral load testing in all the sites. In such case, management of ART program through CD4 count would be effective in achieving the goal to control the progression of HIV in Nepal.

In the past decade, a number of studies have sought to determine the reference range of CD4 and CD3 count in different parts of the world [10]. The variation in reference range due to climatic factors, geographical locations, dietary habits, ethnic variation and environmental factors in different studies has already been documented [11–13]. Nepal is a country with wide geographical, climatic and ethnic variation. A study carried out in Eastern, Central and Western Development region of Nepal showed different mean values for CD4 count [1]. Because Nepal has a wide ethnic and climatic variation, regional as well as national reference range seems important. Reference range of CD4 had been established in Eastern, Central and Western development region of Nepal while the same is still to be established in Mid-western and Far-western development region of the country. This study was carried out with the objective of establishing regional reference range of CD4 T lymphocyte count as well as national reference range with the help of data from previous study in Nepal.

## Main text

### Methods

A descriptive cross-sectional study was carried out among healthy adult population in mid-western and far-western development region of Nepal from March to May 2016. Samples were collected from healthy volunteers willing to participate in the study after filling questionnaire form. The form was filled by face-to-face interview for each participant. The basic information included in the form was: name, age, sex and, history of acute or chronic disease. Only the healthy adults of 15–60 years with no previous history of any kind of diseases, recent medication or blood transfusion were included in the study. Women in the pregnancy period were excluded from the study (Additional file 1). The collected samples were tested for the presence of HIV antibodies. Among the HIV sero-negative healthy volunteers, 400 were selected for study (200 in each site). The selected samples were processed for further laboratory analysis at two major government based hospitals; Bheri zonal hospital and Seti zonal hospital. The sample size was in accordance to the CLSI guidelines for determining laboratory reference ranges which indicates a minimum of 153 subjects to be enrolled in the study for 95th percentile value with 95% confidence interval [14]. The sample was collected in between 10.00 AM to 2.00 PM to minimize the diurnal variation. To obtain homogeneity in data, participants from different districts of the region were included. In addition, enrollment of both sex, and adults from different age group were also considered to eliminate the possible bias.

About 3 ml whole blood sample was collected in tripotassium ethylenediaminetetraacetic acid (K3EDTA) vial from the healthy volunteers participating in study. All the samples were tested for the presence of HIV antibodies following national algorithm that includes HIV1/2 rapid test assay using three different commercial kits: Determine, Stat-Pak and Uni-Gold. HIV negative samples were assayed for CD3 and CD4 T lymphocyte count.

T lymphocyte count was carried out using BD FACS Calibur (BD Biosciences, San Jose, CA, USA) following manufacturer’s instructions. Briefly, 50 μl of well-mixed EDTA whole blood was pipetted into a BD Trucount tube containing bead. 20 μl of BD Tritest™ CD3/CD4/CD45 reagent containing monoclonal antibodies for CD3, CD4 and CD45, labeled with fluorescent dyes was also added to the same tube. The tube was then vortexed, and incubated in dark at room temperature for 15 min to stain the cells. Following staining, red blood cells were lysed using BD FACS lysing solution and run in the flow cytometer to obtain absolute count.

For the purpose of quality control, calibration beads were run every day to ensure the optical alignment of the instrument following compensation setting using the software. Internal quality control was also run at both the centers using commercially available stabilized blood samples. Samples were run only when the result of internal quality control was within the range of the value provided by the manufacturer.

Data collected were initially entered into MS Excel, which was then transferred to Statistical Package for Social Sciences (SPSS) and analyzed using the same SPSS version 20.0 [IBM Armonk, NY, USA]. Mean, median and standard deviation values were calculated for each parameter. Wilks-Shapiro test was used to understand the distribution of data, which showed significant difference suggesting non-Gaussian distribution. Based on this, the decision for non-parametric analysis was made to establish the reference range. The reference range was defined as the central 95% of the area under the distribution curve of the values (from 2.5% to 97.5%). ANOVA test was used to compare mean between variables considering 95% CI and, p value less than 0.05 was considered statistically significant.

## Results

A total of 400 healthy adults (157 females and 243 males) were enrolled in study with the ratio of female to male, 1:1.5. Age of the participants included in the study range from 15–60 years with the mean age of 27 years. The overall mean, standard deviation and 95% CI were obtained for both CD4 and CD3 T lymphocyte. The data showed that both the CD4 (p-value 0.000) and CD3 (p-value 0.000) values were significantly different in male compared to female. Male had lower value for both CD4 (757 ± 270) and CD3 (1465 ± 499) in comparison to female, CD4 (914 ± 303) and CD3 (1671 ± 560) (Table 1).

**Table 1:** T lymphocytes subset in different age groups and sex (N = 400)

Variables	Mean ± SD	SE	95% CI	Minimum	Maximum	p-value
CD3						
Sex						
Male	1465 ± 499	32.0	1402–1528	510	3091	*0.000*
Female	1671 ± 560	44.7	1582–1759	635	3737	
Age group						
15–29	1549 ± 493	29.2	1492–1607	510	3228	0.960
30–44	1533 ± 637	64.6	1404–1661	612	3737	
45–59	1559 ± 544	124.8	1297–1822	675	2888	
Sub-total	1546 ± 532	26.6	1494–1598	510	3737	
CD4						
Sex						
Male	757 ± 270	17.3	723–791	310	1754	*0.000*
Female	914 ± 303	24.2	867–962	306	1917	
Age group						
15–29	817 ± 280	16.6	784–850	306	1756	0.795
30–44	814 ± 327	33.1	749–881	328	1917	
45–59	863 ± 328	75.3	705–1021	376	1754	
Sub-total	819 ± 294	14.7	790–848	306	1917	

Most of the participants had absolute CD4 T lymphocyte count in between 500 and 1000 cells/μl (65.25%) followed by 1000–1500 cells/μl (18.5%), < 500 cells/μl (13.0%) and > 1500 cells/μl (3.25%). The reference range for CD4 (380–1553 cells/μl) and CD3 (743–2803 cells/μl) were determined in participants from western Nepal. Because male and female had significantly different mean values for both CD4 and CD3 T lymphocyte, the reference range was determined separately for male and female. Reference range of CD4 was (344–1470) cells/μl for male and (393–1599) cells/μl for female. Likewise, reference range of CD3 was (753–2762) cells/μl for male and (737–3241) cells/μl for female. In addition, reference range for CD4 and CD3 were also calculated separately in Mid-western development region and Far-western development region of the country (Table 2). The national reference range of CD4 T lymphocyte was determined to be (798 ± 335) cells/μl (Table 3).

**Table 2:** Absolute CD4 count in healthy Nepalese adult from western Nepal

	Mid-western region (N = 200)			Far-western region (N = 200)			Total (N = 400)		
	Mean±SD	Median	2.5th –97.5th Percentile	Mean±SD	Median	2.5th–97.5th Percentile	Mean±SD	Median	2.5th –97.5th Percentile
CD4									
Male	744 ± 274	689	337–1626	768 ± 268	736	386–1467	757 ± 270	716	344–1470
Female	830 ± 267	815	388–1546	1014 ± 315	969	467–1784	914 ± 303	870	393–1599
Sub-total	780 ± 273	753	341–1552	857 ± 309	809	396–1560	819 ± 294	785	380–1553
CD3									
Male	1427 ± 492	1347	676–2794	1500 ± 504	1410	794–2754	1465 ± 499	1381	753–2762
Female	1481 ± 414	1491	699–2387	1894 ± 626	1857	904–3532	1671 ± 560	1562	737–3241
Sub-total	1450 ± 460	1390	693–2523	1642 ± 581	1526	820–3114	1546 ± 532	1468	743–2803

**Table 3:** Mean CD4 count in normal healthy individuals from different countries/population

Countries/population	Absolute CD4 (Mean ± SD)	References
Mexico	(800 ± 230) cells/μl	[16]
Indonesia	(753 ± 270) cells/μl	[17]
England	(830 ± 290) cells/μl	[18]
Nigeria	(847 ± 307) cells/μl	[19]
Tanzania	(745 ± 256) cells/μl	[11]
India	(919 ± 311) cells/μl	[10]
Turkey	(1095 ± 391) cells/μl	[21]
South Indians	1048 cells/μl	[20]
China	(727 ± 255) cells/μl	[2]
Ethiopia	660,775 (Median)	[22, 28]
Uganda	836, 838,1256 (Median)	[22, 24]
Nepal		
Eastern	715 ± 234	[1]
Central	835 ± 306	
Western	807 ± 167	
Mid-western region	780 ± 273	Present study
Far-western region	857 ± 309	
National reference value	798 ± 335	Present study and (1)

## Discussion

CD4 T lymphocyte is an essential marker in guiding clinical decisions regarding disease staging and therapeutic monitoring in case of HIV infection [6, 15]. In order to establish the reference range of T lymphocyte count employed, unemployed and students of adult age group were selected from different districts without bias. Furthermore, participants from different ethnic groups, geographical area and gender were also enrolled to eliminate the possible bias.

The mean value of absolute CD4 count obtained in this study, (819 ± 294) cells/μl is comparable to the previous study by Shakya et al. carried out in three development regions of Nepal [1]. The result is also similar to the studies from Mexico city (800 ± 230), Indonesia (753 ± 270), England (830 ± 290), Nigeria (847 ± 307) and Tanzania (745 ± 256) [11, 16–19]. However, the CD4 mean value obtained in our study is comparatively lower than those from India (919 ± 311), Turkey (1095 ± 391) and South India (1048) and, higher than Chinese adults (727 ± 255) [2, 10, 20, 21] (Table 3). The Saharan African population had a diverse distribution of CD4 counts from Ethiopia (660) to Uganda (1256) [22–24]. The deviation of mean CD4 count in population from different geographical area might be due to different methodology employed for the study. Also, it seems likely that these results are in fact due to multiple factors such as lifestyle and ethnic variations in populations from different parts of the world.

The present study obtained higher value of CD4 and CD3 T lymphocyte for female compared to male. This is comparable to that of the previous study by Shakya et al. in three development region (Eastern, Central and Western) of Nepal [1]. The higher CD4 count among female was also documented by the study of Grinsztejn et al. and Lee et al. [25, 26]. The possible explanation for such a result might be the influence of sex hormones on immunity that is responsible for higher CD4 count in female [22, 27]. Since the present study obtained significantly different values of absolute CD4 and CD3 T lymphocyte count in male and female population, it was felt appropriate to establish separate reference range for male and female. The reference range of CD4 T lymphocyte was determined to be (344–1470) cells/μl for male and (393–1599) cells/μl for female. Similarly, the reference range of CD3 T lymphocyte was calculated to be (753–2762) cells/μl for male and (737–3241) cells/μl for female.

There are large numbers of ethnic groups residing throughout the country with different socio-economic status. Few ethnic groups are found living in particular geographical area and have distinct lifestyle. A previous study in 2012 by Shakya et al. established the reference range in Eastern, Central and Western development region of the country [1]. This study has determined reference range in remaining two development regions; Mid-western and Far-western development regions. When we combine the data from the study of Shakya et al. and the present study, we obtain the mean average absolute CD4 T cell count 798 ± 335 cells/μl that can be taken as the national reference value for Nepalese healthy adults (Table 3).

## Conclusion

The study established the reference range of CD4 and CD3 T lymphocyte count in healthy adults from western part of Nepal and determined the national reference value of CD4 T cell. It also suggests using separate values of reference range for male and female population.

## Limitation

We combined the result of two study carried out at different interval of time; first in year 2009 and the next in year 2016 to include the data from different parts of the country for establishment of national reference range of CD4 T lymphocyte count.

## Supplementary information

**Additional file 1:** Questionnaire form for participating individuals. The data in the form is a set of questions to be asked by a health worker to the participating volunteers to access their health status along with other information.

## Data Availability

The raw data and materials of this work would be made available to the concerned person upon request.

## Abbreviations

ART: Antiretroviral therapy
HIV: Human immunodeficiency virus
CD: Cluster of Differentiation
RNA: Ribonucleic Acid
CLSI: Clinical and Laboratory Standards Institute
EDTA: Ethylenediaminetetraacetic acid
NPHL: National Public Health Laboratory
