# Supplementary material for: Establishment of reference range of CD4 T-lymphocyte in healthy Nepalese adults
Source: BMC Res Notes. 2020 Jul 2;13:316. doi: 10.1186/s13104-020-05156-5 (PMC7330941; doi:10.1186/s13104-020-05156-5)
Supplement: Supplementary file 1 — Additional file 1: Questionnaire form for participating individuals. The data in the form is a set of questions to be asked by a health worker to the participating volunteers to access their health status along with other information. [file 13104_2020_5156_MOESM1_ESM.pdf]

Government of Nepal
Ministry of Health and Population
Department of Health Services

National Public Health Laboratory

Teku, Kathmandu

Questionnaire Form for Participating Volunteers

Project Title: Establishment of Reference Range of CD4 T Lymphocyte

Site: **Form No. :**

Instruction to the Health Worker: Please make sure that the consent form is signed before administering this questionnaire. Furthermore, make sure that the individual is eligible to be included in the study before proceeding with the following data collection. Take note of these eligibility requirements:
Inclusion Criteria: Apparently healthy adults with no history of any acute or chronic disease
Exclusion Criteria: Healthy individual with age below 15 years or above 60 years. Women in the pregnancy period.
Read to the volunteers: Thank you for agreeing to participate. I will ask you few questions. Please answer truthfully. Your answers to these questions will be kept confidential at all times.

Demographic Information

Surname: Occupation:

Age: Sex:

Address:

Clinical Information

Any history of acute disease in past 3 months: ☐ Yes ☐ No

If yes, Name of disease: ……………………………………………………….

Medication taken: ……………………………………………………………

History of any chronic disease: ☐ Yes ☐ No

Recent Medication if any: ……………………………………………………….

Blood transfusion in past 3 months: ☐ Yes ☐ No

Laboratory Investigation

HIV status: ☐ Positive ☐ Negative

WBC count:

Differential Leukocyte Count: N…………… L…………. M………… E…………... B……….

CD45 count: …………… CD3 count: ……………..

CD4 count: …………….
